# The prevalence of pain at pressure areas and pressure ulcers in hospitalised patients

**DOI:** 10.1186/1472-6955-12-19

**Published:** 2013-07-31

**Authors:** Michelle Briggs, Michelle Collinson, Lyn Wilson, Carly Rivers, Elizabeth McGinnis, Carol Dealey, Julia Brown, Susanne Coleman, Nikki Stubbs, Rebecca Stevenson, E Andrea Nelson, Jane Nixon

**Affiliations:** 1 Centre for Pain Research, Queens Square House, Leeds Metropolitan University, Leeds LS1 3HE, UK; 2 Clinical Trials Research Unit, Leeds Institute of Clinical Trials Research, University of Leeds, Leeds LS2 9JT, UK; 3 Rick Hansen Institute, 6400-818 West 10th Avenue, Vancouver, BC V5Z 1M9, Canada; 4 Leeds Teaching Hospital NHS Trust, Leeds, UK, Great George Street, Leeds LS1 3EX, UK; 5 Clinical Research Unit, Old Nuclear Medicine, Research & Development - University Hospitals Birmingham NHS Foundation Trust, Queen Elizabeth Hospital, Queen Elizabeth Medical Centre, 1st Floor Pharmacy Building, Room 3, Birmingham B15 2TH, UK; 6 Tissue Viability, St Mary’s Hospital, Greenhill Road, Armley LS12 3QE, UK; 7 School of Healthcare, University of Leeds, Room 1.25, Baines Wing, Leeds LS2 9JT, UK

**Keywords:** Pain, Pressure ulcers, Risk assessment, Prevalence

## Abstract

**Background:**

Patients with pressure ulcers (PUs) report that pain is their most distressing symptom, but there are few PU pain prevalence studies. We sought to estimate the prevalence of unattributed pressure area related pain (UPAR pain) which was defined as pain, soreness or discomfort reported by patients, on an “at risk” or PU skin site, reported at a patient level.

**Methods:**

We undertook pain prevalence surveys in 2 large UK teaching hospital NHS Trusts (6 hospitals) and a district general hospital NHS Trust (3 hospitals) during their routine annual PU prevalence audits. The hospitals provide secondary and tertiary care beds in acute and elective surgery, trauma and orthopaedics, burns, medicine, elderly medicine, oncology and rehabilitation. Anonymised individual patient data were recorded by the ward nurse and PU prevalence team. The analysis of this prevalence survey included data summaries; no inferential statistical testing was planned or undertaken. Percentages were calculated using the total number of patients from the relevant population as the denominator (i.e. including all patients with missing data for that variable).

**Results:**

A total of 3,397 patients in 9 acute hospitals were included in routine PU prevalence audits and, of these, 2010 (59.2%) patients participated in the pain prevalence study. UPAR pain prevalence was 16.3% (327/2010). 1769 patients had no PUs and of these 223 patients reported UPAR pain, a prevalence of 12.6%. Of the 241 people with pressure ulcers, 104 patients reported pain, a UPAR pain prevalence of 43.2% (104/241).

**Conclusion:**

One in six people in acute hospitals experience UPAR pain on ‘at risk’ or PU skin sites; one in every 8 people without PUs and, more than 2 out of every five people with PUs. The results provide a clear indication that all patients should be asked if they have pain at pressure areas even when they do not have a PU.

## Background

A pressure ulcer (PU) is a “localised injury to the skin and/or underlying tissue usually over a bony prominence, as a result of pressure, or pressure in combination with shear” and they range in severity from skin redness (Category 1) and superficial skin loss (Category 2) to severe ulcers involving fat, muscle and bone (Category 3, 4 or unstageable) as defined by the European Pressure Ulcer Advisory Panel and National Pressure Ulcer Advisory Panel [1]. These wounds are prevalent, can occur in all healthcare settings and represent a worldwide challenge, as they are associated with high healthcare and personal costs [2-5].

Pain has been reported by patients to be a major symptom associated with PUs, with dressing changes being particularly painful times [6]. PU pain can be debilitating, reducing the individual’s ability to participate in physical and social activities, adopt comfortable positions, move, walk, and under-go rehabilitation. People with PU pain describe their experience as “endless pain” characterised by constant presence, needing to keep still, equipment and treatment pain [3,7,8]. The desire of healthcare professionals to understand the extent of this problem is demonstrated by a number of reviews relevant to the topic of pain and pressure ulcers [5,6,9,10].

Reviews of the literature carried out by Girouard et al., 2008, and Pieper et al. 2009 described how PU pain had been measured in a number of studies [9,10]. They also synthesised research on the prevalence of PU pain. The two reviews identify 8 studies reporting the prevalence of pain associated with pressure ulcers in samples ranging from 20 to 186 participants in diverse populations including hospital, community and palliative care. In the two largest studies (>100 participants), pressure ulcer pain prevalence estimates were 37% and 66%. Data quality is an issue as only 5 studies, however, used validated and reliable measures to assess pain. Furthermore, the methods of pain assessment differed, for example, some studies described nurse reported pain where nurses are asked to judge how much pain a patient is experiencing as opposed to direct patient reported outcomes. The former has been shown to result in under-reporting of patients’ pain in other situations [11].

Gorecki et al., (2011) carried out a mixed methods systematic review [6] and identified and synthesised qualitative and quantitative studies of patients’ reports of PU pain. Ten studies were included (6 qualitative and 4 quantitative) representing the experience of 108 adults with PUs. The PU pain experience was mapped, producing a conceptual framework of five domains: communicating the pain, feeling the pain, impact of pain, self-management, and professional management. The review concluded that improved communication of pain was needed between the individual and health care professionals to promote more effective PU pain management in the future.

However, the Gorecki et al., (2011) review was limited because they were unable to evaluate PU descriptors for Category 1 PUs (the most prevalent PU Category) [6]. A problem with research in this field is that there is a paucity of research about pain associated with Category 1 PUs. Only one patient from the combined review sample had a Category 1 PU, the majority of patients had multiple PUs of mixed categories. Furthermore, the systematic review of patients’ experiences of pain and pressure ulceration highlighted that pain at skin sites was experienced by patients prior to PU development but was often not recognised as important by their health care professionals [3,6]. Patients felt that they were responsible for communicating pain and that their care provider was responsible for attending to it, but patients’ views and concerns did not always prompt action and many healthcare professionals dismissed patients’ reports of pain at pressure areas [6,12].

In order to describe pain in patient populations with and without PUs, we developed three definitions as follows:

UPAR pain: Unattributed Pressure Area Related pain which is defined as pain, soreness or discomfort on any “at risk” or PU skin site, reported at a patient level.

PAR pain: Pressure Area Related pain which is defined as pain, soreness or discomfort on any “at risk” skin site, reported at a skin site level (i.e. attributable).

PU pain: Pressure Ulcer pain which is defined as pain, soreness or discomfort on any PU skin site, reported at a skin site level (i.e. attributable).

The first in a series, this study sought to estimate the prevalence of unattributed pressure area related pain (UPAR pain) in hospital populations in a large representative sample in order to provide definitive estimates. This study is part of a suite of 6 studies which comprise the Pressure UlceR Programme of ReSEarch (PURPOSE), funded by the National Institute for Health Research (NIHR *PG-0407-10056)* which aims to reduce the impact of PUs on patients through improved risk assessment and the development of measures to capture patient reported outcomes.

### Objectives

1. To estimate an overall UPAR pain point prevalence in an acute hospital population

2. To estimate the point prevalence of UPAR pain in patients without PUs

3. To estimate the point prevalence of UPAR pain in patients with PUs.

## Methods

### Study design

We undertook a multi-centre, cross-sectional study in 9 hospitals across 2 large teaching NHS Trusts (6 hospitals) and 1 district general hospital NHS Trust (3 hospitals) in England to establish UPAR pain point prevalence. Questions about pain were added to the routine annual PU prevalence audits undertaken in the participating NHS Trusts. In addition to the standard PU data recorded by ward and audit teams, patients were asked two questions relating to pain at pressure areas by a member of the Tissue Viability Team. These questions were

1. At any time, do you get pain, soreness or discomfort at a pressure area (prompt: back, bottom, heels, elbows or other as appropriate to the patient)

2. Do you think this is related to either; your pressure ulcer OR lying in bed for a long time OR sitting for a long time?

The data were asked at a global patient level, and not by skin site, hence the pressure area related pain is not attributable to individual skin sites with or without PUs. These questions were adapted from the case screening questions used in a large postal survey of pain prevalence in the UK [13]. Data were collected on one identified day in each Trust during 2009 -2010.

### Eligibility criteria

As per standard PU prevalence audit methodology, the target population was “all inpatients of 18 years of age or older who were in hospital on the date of the participating Trust’s PU prevalence audit”. Patients in paediatric, obstetric and psychiatric care settings were excluded from the pain survey, as the prevalence of PU in these settings is very low, and hence the data collection to information burden ratio is unacceptably high in these settings. In addition to the standard PU audit data, the ward nurse was asked to consider whether each patient was able to report the presence or absence of pain.

Patients were excluded from the pain prevalence study where it was considered ethically or clinically inappropriate by the ward nurse/clinical team, for example, those where death was considered to be imminent. Where patients were assessed as able to report pain these patients were eligible for inclusion in the pain prevalence. Where patients were assessed as not able to report pain this was recorded together with the reasons for ineligibility.

### Data collection

Standard practice for the PU prevalence audit was used to assess and record data to ensure data capture for the total hospital population. Anonymised individual patient data was recorded by a designated ward nurse trained in the use of the data collection form and skin assessment as part of the preparation for the audit. Data recorded included ward speciality, date of birth, gender, height, weight, ethnicity, mobility, risk assessment scale (as per local policy) and skin classification by skin site using the European Pressure Ulcer Advisory Panel classification [14]. The EPUAP 1998 classification (and not the revised EPUAP/NPUAP 2009 version) [1] was used as the 1998 version was still in use at the participating sites. The classification was adapted for audit and research use to enable confirmation of normal skin and unstageable PUs. Where a patient had no limb or a chronic wound these were recorded as N/A and ‘other chronic wound’ respectively for that skin site. The audit forms were checked by a member of the audit team and where it was indicated that the patient was well and able to report pain, a member of the Tissue Viability Team asked the patient the two pain questions.

### Analysis

The analysis of this prevalence survey included data summaries and no inferential statistical testing was planned or undertaken. Percentages were calculated using the total number of patients from the relevant population as the denominator (i.e. including all patients with missing data for that variable). All analyses were carried out using SAS software.

### Ethical approval

The study was approved by the Leeds Central Research Ethics Committee (REC) prior to data collection. Ref No 09/H1313/14.

## Results

**Figure 1:**
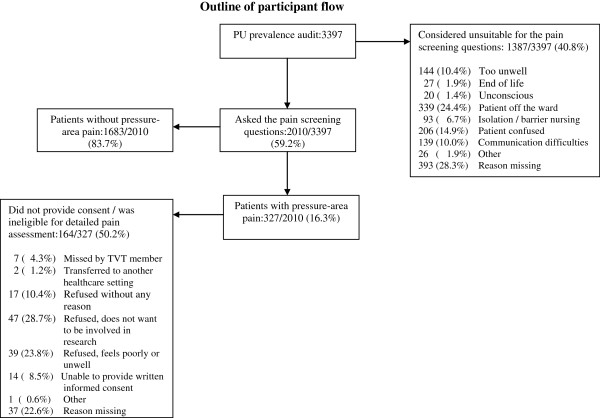
Outline of participant flow.

Figure 1 describes the flow of participants through each stage of the process.

### Total hospital population

A total of 3,397 patients in 9 acute hospitals were included in the routine PU prevalence surveys and this constitutes our target hospital population. The mean age of patients was 65.8 (SD 19.23), range 18-103 years. The numbers of men and women were similar: 48.7% male (1655/3397), and 50.9% female (1730/3397). The number of patients audited by place of assessment is presented in Table 1.

**Table 1:** Total hospital population by place of assessment

**No. of participants**	**%**	
Medicine	1348	39.7%
Surgery	868	25.6%
Elderly medicine	380	11.2%
Orthopaedic and trauma	305	9.0%
Oncology	211	6.2%
Critical care	179	5.3%
Rehabilitation	79	2.3%
Burns	15	0.4%
Clinical decision units	8	0.2%
Missing	4	0.1%
Total	3397	100%

Of the 3,397 participants included in the routine PU prevalence, 502 (14.8%) patients were reported to have 1066 PUs (mean 2.1 per patient, SD 1.63, range 1-13). The majority (70.5%; 752/1066) of reported PUs were Grade 1, 22.2% (237/1066) were Grade 2 and less than 1 in 10 (7.2%; 77/1066) were severe PUs.

### Pain prevalence population

Of the 3,397 hospital patients in the PU audit sample, 2010 (59.2%) were considered well enough to respond to the pain questions and hence were eligible for the pain prevalence. Of the 2010 patients in the pain prevalence population, 241/2010 (12.0%) had one or more PU. The PU profile of the categories observed from the total hospital population and pain prevalence population are detailed in Table 2, illustrating that there were similar Grades of PU in both populations.

**Table 2:** PU prevalence for total and pain prevalence populations

**Grade**	**Total hospital**	**Pain prevalence**
	**prevalence**	**population**
Total Population	3,397	2010
Number of patients with PU	502 (14.8%)	241 (12.0%)
Total number of PUs	1066	491
Grade of PUs reported		
Grade 1	752 (70.5%)	357 (72.7 %)
Grade 2	237 (22.2%)	100 (20.4%)
Grade 3	45 (4.2%)	18 (3.7%)
Grade 4	18 (1.7%)	10 (2.0%)
Unstageable	14 (1.3%)	6 (1.2%)

### Pain prevalence rates

Of the 2010 people asked the pain questions, 327 said yes to both questions, indicating they had pain on one or more skin sites with or without a PU, providing an overall UPAR pain prevalence of 16.3%. 1769 patients had no PU and of these 223 patients reported pain, an UPAR pain prevalence of 12.6%. Of the 241 people with PUs, 104 patients reported pain at one or more PU site, an UPAR pain prevalence of 43.2% (104/241).

## Discussion

This study is the first to assess UPAR pain in a large representative hospital population including patients with and without PUs. The importance of inclusion of patients without PUs is underlined by the findings that 12.6% of patients without PUs reported pain on an ‘at risk’ skin site (one in eight people without obvious tissue damage). This group could be important because they are reporting pain over an ‘at risk’ skin site which they believed to be caused by pressure but they were not yet displaying damage clinically. There are no other published reports of this type of pain.

In light of this finding, there could be gains from improving the recognition and treatment of pain in the ‘at risk’ population. The relationship between pressure area related pain and pressure injury is likely to be complex and this prevalence study was not designed to explain this relationship, rather it provides descriptive data to inform further study. As our pain prevalence study was cross-sectional we are not able to determine whether pain on an ‘at risk’ skin site is an early indicator of PU development; a prospective cohort study is currently underway addressing this research question.

In relation to UPAR pain reported by patients with PUs, 43.2% of patients reported pain. It is important to note that the pain reported in this study is not necessarily PU pain, since the pain reports were not attributable to a particular PU, rather any “at risk” or PU skin site irrespective of damage. However, the pain prevalence is more than 3-fold the prevalence found in patients without PUs and the prevalence rate is comparable to the prevalence of pain in other chronic wounds in European populations [15,16]. Furthermore, evidence suggests that the pain management for this group is not comparable to other painful conditions such as musculoskeletal pain. For example, in a study of people with PUs, 59% of the sample reported PU pain but only 2% received analgesia within 4 hours of the interview [17]. Quirino and colleagues in a small study of 20 patients noted that pain compromised movement and that 80% of patients had pain for more than 1 hour a day [18]. Fourteen patients in their study reported use of analgesia (mainly NSAIDS) but reported little or no effect.

There is a need to improve the assessment and treatment of pain for people with PUs as this will affect nearly half of the PU population based on our findings. There is also a need to provide pre-emptive treatment when pain due to pressure can be predicted. For example, in a study which investigated the pain associated with turning for pressure relief and other nursing procedures, turning was the most painful procedure compared to tracheal suction, wound drain removal, central line insertion and femoral sheath removal [19]. It was noted that despite this painful procedure, patients rarely received analgesia prior to turning.

The findings of this study underline the importance of assessing all patients for the presence/absence of pain and this should be a fundamental component of pressure area care. A key recommendation from Gorecki et al., 2011 was that we need to develop better methods of communicating about pain in relation to PU development [6]. This study adds further information to guide clinicians and researchers in this regard.

### Methodological limitations

Point prevalence rates provide evidence of the scale of a clinical problem and the general limitations of cross-sectional studies are acknowledged [4,20-22]. Other limitations of this study are that skin assessment data was recorded by clinical staff which has inherent limitations [8,23,24] which may have resulted in over or under-reporting of PUs or misclassification of Grade or extent of tissue damage, particularly at Grade 1, which is prone to misclassification. The pain was recorded at the patient level and not by skin site and so it was not possible to assess the level of PU pain. We were not able to record pain treatment and therefore the quality of pain management may differ between wards and could be an additional factor to consider Furthermore, the methodology used meant that a significant proportion of hospital patients (40.8%) were not able to participate in the pain prevalence study due to illness (too unwell, end of life, unconscious), difficulty in assessing (confused or communication difficulty) or patient unavailable (off the ward or in isolation). The overall PU prevalence in the hospital population is higher than the pain prevalence population suggesting that the overall UPAR pain prevalence of 16.3% may be an under-estimate and generalisability is limited by our inability to assess really unwell patients and those with communication difficulties. Therefore this limits the generalisability to a total hospital population.

## Conclusion

The results provide a clear indication for the implementation of monitoring pressure area related pain, and pain management in patients with and without PU. This is in an area which is a priority for patients and impacts upon the quality of life of nearly half of the population. More people with PUs reported UPAR pain than those without, however an important minority of patients without PUs reported UPAR pain. A prospective cohort study is currently underway to provide further insights into the relationship between pain and subsequent Category 2 PU development.

This article presents independent research commissioned by the National Institute for Health Research (NIHR) under its Programme Grants for Applied Research funding scheme (RP-PG-0407-10056). The views expressed in this article are those of the authors and not necessarily those of the NHS, the NIHR or the Department of Health.

## Competing interests

The authors declare that they have no conflict of interest.

## Authors’ contributions

MB contributed to the conception and design of the study, analysis and interpretation of the results and drafted the manuscript. MC contributed to the design of the study, performed the statistical analysis and provided revision to the draft manuscript. LW co-ordinated the data collection, contributed to the interpretation of the results and revising the manuscript. CR contributed to the design of the study and revising the manuscript. EMcG contributed to the conception and design of the study, the interpretation of the results and revising the manuscript. CD contributed to the conception, design of the study, the interpretation of the results and revising the manuscript. NS contributed to the conception and design of the study, the interpretation of the results and revising the manuscript. JB contributed to the concept and design of the study and interpretation of the results. SC co-ordinated the data collection, contributed to the interpretation of the results and revising the manuscript. RS contributed to the data collection, the interpretation of the results and revising the manuscript. EAN contributed to the conception, design of the study, interpretation of the results and revising the manuscript. JN conceived of the study, and participated in its design, was responsible for coordination and delivery of the study, interpretation of results and revising the manuscript. All authors read and approved the final manuscript.

## Pre-publication history

The pre-publication history for this paper can be accessed here:

http://www.biomedcentral.com/1472-6955/12/19/prepub
